# Association between echocardiography-derived haemodynamic force parameters and left ventricular reverse remodelling after cardiac resynchronization therapy

**DOI:** 10.1093/ehjci/jeae181

**Published:** 2024-07-17

**Authors:** Dorien Laenens, Pieter van der Bijl, Xavier Galloo, Alessandro C Rossi, Giovanni Tonti, Johan H C Reiber, Gianni Pedrizzetti, Nina Ajmone Marsan, Jeroen J Bax

**Affiliations:** Department of Cardiology, Leiden University Medical Centre, Albinusdreef 2, 2333 ZA Leiden, The Netherlands; Department of Cardiology, Leiden University Medical Centre, Albinusdreef 2, 2333 ZA Leiden, The Netherlands; Department of Cardiology, Leiden University Medical Centre, Albinusdreef 2, 2333 ZA Leiden, The Netherlands; Department of Cardiology, University Hospital Brussels, Vrije Universiteit Brussel, Laarbeeklaan 101, 1090 Brussels, Belgium; Medis Medical Imaging, Schuttersveld 9, 2316 XG Leiden, The Netherlands; Cardiology Division, G. D’Annunzio University, Chieti, Italy; Medis Medical Imaging, Schuttersveld 9, 2316 XG Leiden, The Netherlands; Department of Radiology, Leiden University Medical Centre, Albinusdreef 2, 2333 ZA Leiden, The Netherlands; Department of Engineering and Architecture, University of Trieste, Via Alfonso Valerio, 6/1, 34127 Trieste TS, Italy; Department of Biomedical Engineering, University of California, 402 E Peltason Dr, Irvine, CA 92617, USA; Department of Cardiology, Leiden University Medical Centre, Albinusdreef 2, 2333 ZA Leiden, The Netherlands; Department of Cardiology, Leiden University Medical Centre, Albinusdreef 2, 2333 ZA Leiden, The Netherlands; Department of Cardiology, Turku Heart Center, University of Turku and Turku University Hospital, Kiinamyllynkatu 4-8, 20521 Turku, Finland

**Keywords:** fluid dynamics, haemodynamic forces, cardiac resynchronization therapy, left ventricular reverse remodelling

## Abstract

**Aims:**

Cardiac resynchronization therapy (CRT) may induce left ventricular (LV) reverse remodelling (=LV response) in patients with heart failure. Intraventricular pressure gradients can be quantified using echocardiography-derived haemodynamic forces (HDF). The aim was to evaluate the association between baseline HDF and LV response and to compare the change of HDF after CRT between LV responders and LV non-responders.

**Methods and results:**

The following HDF parameters were assessed: (i) apical–basal (AB) strength, (ii) lateral–septal strength, (iii) force vector angle, (iv) systolic AB impulse, (v) systolic force vector angle. LV response was defined as a reduction of LV end-systolic volume ≥ 15% at six months. One hundred ninety-six patients were included [64 ± 11 years, 122 (62%) men], 136 (69%) showed LV response. On multivariable logistic regression analysis, the force vector angle in the complete heart cycle [OR 1.083 (95% CI: 1.018, 1.153), *P* = 0.012] and the systolic force vector angle [OR 1.089 (95% CI: 1.021, 1.161), *P* = 0.009], both included in separate models, were independently associated with LV response. Six months after CRT, LV responders had greater AB strength, AB impulse, and higher force vector angles, while LV non-responders only showed improvement in the force vector angle in the complete heart cycle.

**Conclusion:**

The orientation of HDF at baseline is associated with LV response to CRT. Six months after CRT, the orientation of HDF improves in LV responders and LV non-responders, while the magnitude of AB HDF only improves in LV responders.

## Graphical Abstract

**Graphical Abstract:**
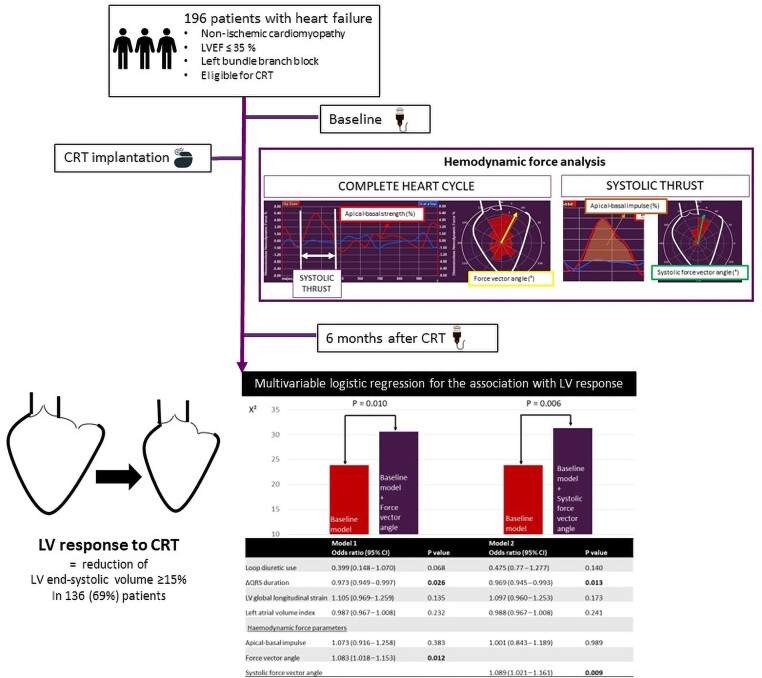

## Introduction

Echocardiography-derived haemodynamic forces (HDF) were recently introduced to quantify pressure gradients in the left ventricle (LV) from routine transthoracic echocardiographic images.^1^ Using speckle tracking technology, dedicated software (QStrain Echo 4.1.4.4., Medis Suite Ultrasound, Medis Medical Imaging, Leiden, The Netherlands) can incorporate LV endocardial motion as well as the mitral and aortic valve area, in a mathematical formula to calculate HDF.^1^ HDF is the result of the interaction of the myocardium, the valves, and the great vessels that enables efficient blood flow. Changes in HDF, and consequently changes in blood flow propulsion and accommodation, represent a first sign of suboptimal LV function before LV remodelling takes place and thereby precede deterioration of LV function.^2^ Accordingly, HDF analysis could be a sensitive tool to detect early changes in LV function in patients with heart failure.

In patients with heart failure, reduced LV ejection fraction (LVEF) and left bundle branch block reduce myocardial contractility and mechanical dyssynchrony, which all negatively affect HDF. Cardiac resynchronization therapy (CRT) has the ability to restore mechanical efficiency of the failing LV in selected patients and can induce LV reverse remodelling.^3^ The occurrence of LV reverse remodelling, often defined as a reduction of LV end-systolic volume (LVESV) ≥ 15%, is associated with improved outcomes after CRT.^4,5^ The link between novel HDF parameters with LV reverse remodelling has not been investigated. In addition, whether HDF is modulated differently after CRT in patients with vs. without LV reverse remodelling is unknown.

Therefore, the aim of the current study was two-fold: (i) to explore the association of baseline HDF parameters (before CRT implantation) with LV reverse remodelling at six months after CRT and (ii) to compare the change of HDF in patients with and without LV reverse remodelling six months after CRT implantation.

## Methods

### Patient population

From a CRT database, patients with heart failure secondary to non-ischaemic cardiomyopathy with reduced LVEF (≤35%), QRS duration ≥ 130 ms, and left bundle branch block were included in this study. Ischaemic cardiomyopathy was an exclusion criterium to avoid the influence of infarcted myocardium (scar) on HDF parameters. The presence of a right bundle branch block was an exclusion criterium, since the hypothesis that a different activation pattern of the ventricles could potentially influence the HDF parameters. Left bundle branch block was defined as a broad, notched or slurred R wave in leads I, aVL, V_5_, and V_6_; absent Q waves in leads I, V_5_, and V_6_ and an R peak time > 60 ms in leads V_5_ and V_6_.^6^ All patients were symptomatic and had class I level A or class IIa level B indications for CRT, according to current heart failure guidelines.^3^ CRT devices were implanted between September 2000 and September 2014 at the Leiden University Medical Center, Leiden, The Netherlands. Patients with suboptimal echocardiographic image quality were excluded from the analysis (see Supplementary data online, *Figure S1*). Image quality assessment was based on frame rate (optimal 50–70 frames per second) and interpretation of the endocardial border tracking. When >1 segment was incorrectly traced, the patient was excluded. Demographic, clinical, electrocardiographic, and echocardiographic data were prospectively collected before CRT implantation in the departmental cardiology information system (EPD-vision; Leiden University Medical Center, Leiden, The Netherlands) and retrospectively analysed. To assess quality of life, the Minnesota Living with Heart Failure Questionnaire was used, while to evaluate exercise capacity, 6 min walking distance was measured. In all patients, transthoracic echocardiography was performed before CRT implantation and at six months follow-up. LV volumes were measured from the apical four- and two-chamber views.^7^ LV reverse remodelling was defined as a reduction in LVESV ≥ 15% at six months.^5^ The study population was dichotomized according to the presence or absence of LV reverse remodelling (i.e. LV responders and LV non-responders). In addition, LV super responders were identified as having a reduction in LVESV ≥ 30% at six months.^5^ The study complies with the Declaration of Helsinki and was approved by the Institutional Review Board. The need for written informed consent was waived by the local ethics committee because of the retrospective design of the study.

### Transthoracic echocardiography

Transthoracic echocardiographic examinations were performed with commercially available ultrasound equipment (Vivid 7 and E9, GE-Vingmed, Horten, Norway). Echocardiographic data were digitally stored for offline analysis using EchoPAC version 203 (GE Medical Systems, Horten, Norway). According to current recommendations, LV and left atrial volumes were measured from the apical four- and two-chamber views.^7^ Simpson’s biplane method was used to calculate LVEF.^7^ Left atrial volume was indexed for body surface area. Speckle tracking strain analysis was performed to calculate LV global longitudinal strain (LV GLS). The region of interest was automatically generated and manually adjusted when required. LV GLS was then calculated by averaging the peak longitudinal strain values of 17 segments, excluding segments that could not be traced correctly. The values of LV GLS are reported as absolute values. The severity of mitral and aortic regurgitation was graded using a multiparametric approach.^8,9^ Moderate and severe mitral/aortic regurgitation were considered significant. Aortic stenosis severity was assessed by aortic valve area, with an area ≤ 1.5 cm² being considered significant.

### Assessment of HDF parameters

**Figure 1:**
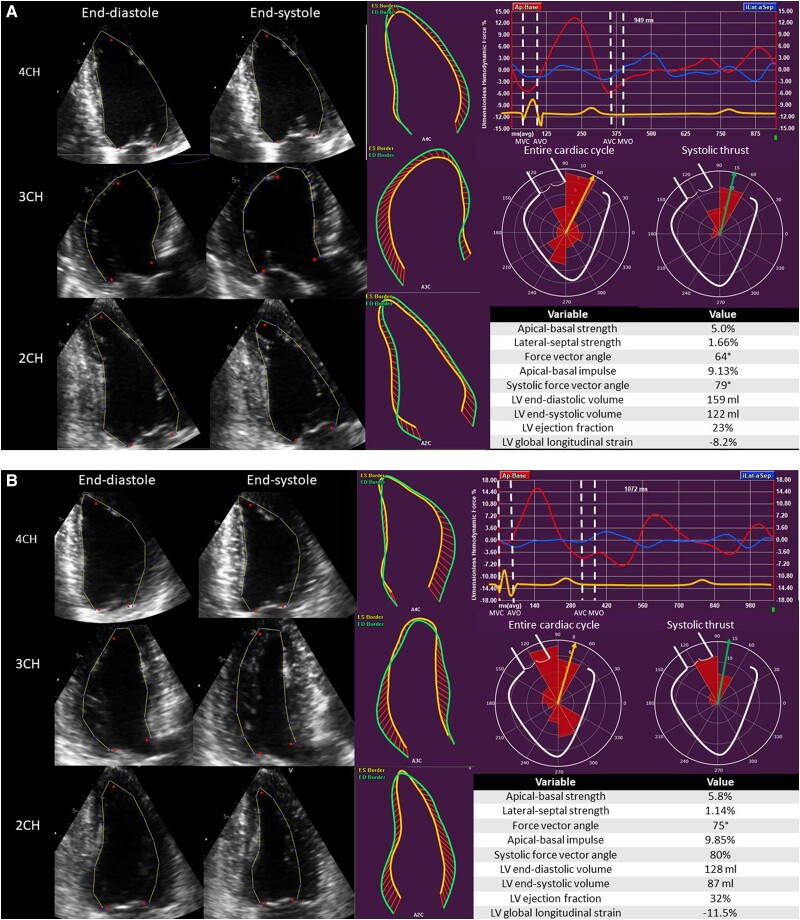
Haemodynamic force analysis in a patient with non-ischaemic cardiomyopathy implanted with a CRT device. (*A*) Before CRT implantation. (*B*) Six months after CRT implantation. 2CH, two-chamber view; 3CH, three-chamber view; 4CH, four-chamber view; AVC, aortic valve closure; AVO, aortic valve opening; LV, left ventricular; MVC, mitral valve closure; MVO, mitral valve opening.

**Figure 2:**
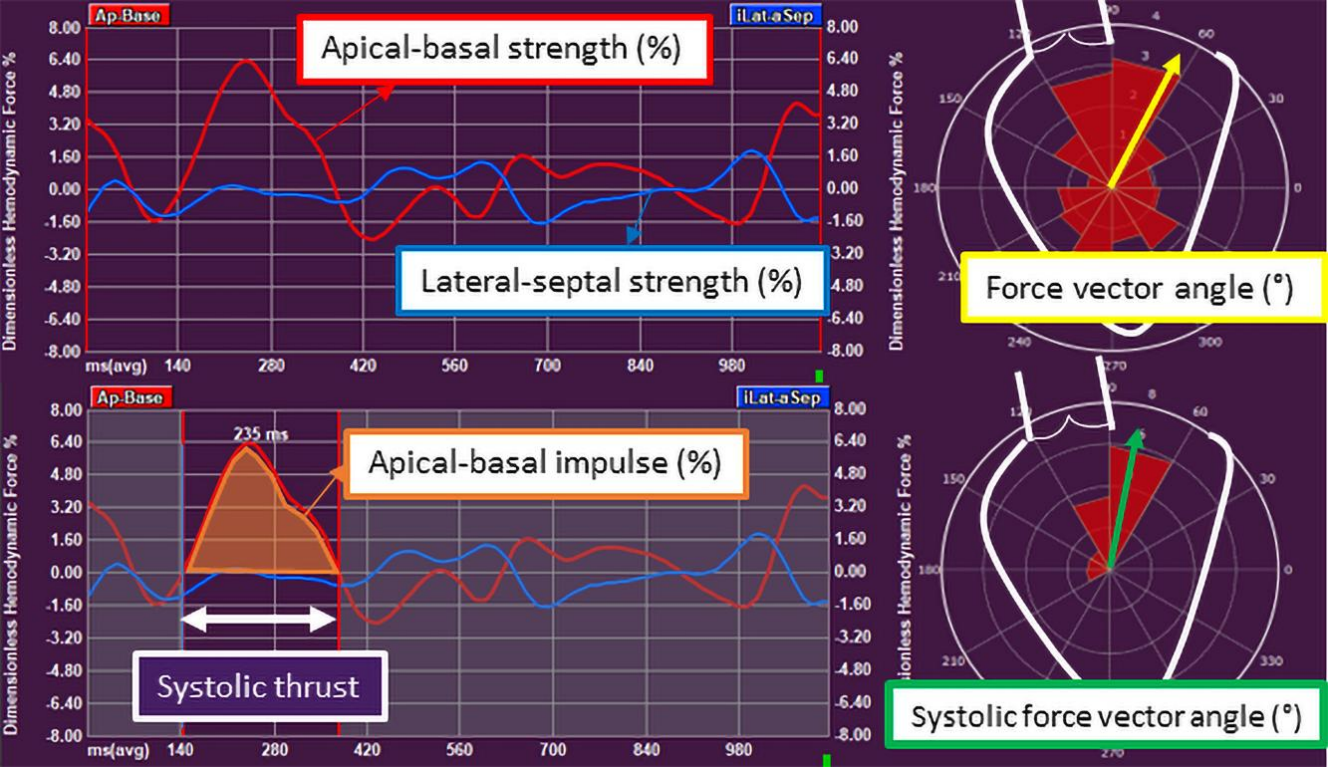
Haemodynamic force parameters in non-ischaemic cardiomyopathy. The apical–basal strength represents the magnitude of force in apical–basal direction. The lateral–septal strength indicates the magnitude of force in lateral–septal direction. The force vector angle of HDF in the complete heart cycle indicates the orientation of HDF. In the systolic thrust, the apical–basal impulse is the area under the curve in the systolic thrust and indicates the magnitude of force in apical–basal direction. The systolic force vector angle represents the orientation of force in the systolic thrust. AVC, aortic valve closure; AVO, aortic valve opening; HDF, haemodynamic forces; LV, left ventricular; MVC, mitral valve closure; MVO, mitral valve opening.

Dedicated software was used to calculate HDF parameters from transthoracic echocardiographic images (QStrain Echo 4.1.4.4., Medis Suite Ultrasound, Medis Medical Imaging, Leiden, The Netherlands). The endocardial border was traced at LV end-systole and LV end-diastole on the four-, three-, and two-chamber apical views (*Figure 1*). Mitral valve area was estimated by measuring the opening of the valve on the apical four-chamber view, while aortic valve opening was estimated using the LV outflow tract diameter, measured from the parasternal long-axis view. The software automatically calculated the HDF parameters using a validated formula.^10^ This formula is based on the velocity at the LV endocardial border and the velocity across the mitral and aortic valves. The following HDF parameters were calculated for the complete heart cycle (*Figure 2*): (i) apical–basal strength, represented as a red curve; (ii) lateral–septal strength, represented as a blue curve, and (iii) the force vector angle, represented as a yellow arrow in the polar histogram. The strength parameters reflect the magnitude of HDF in longitudinal (apical–basal strength) and transverse (lateral–septal strength) directions. The force vector angle reflects the orientation of HDF, with an angle of 90° indicating perfect alignment with the apex–base direction. The amplitude of the apical–basal strength and lateral–septal strength is reported as a root mean square value, including both positive and negative values. The amplitude values are normalized for the LV volume and adjusted for gravity acceleration and fluid density to be expressed as a dimensionless quantity. These parameters are presented as percentages, allowing comparison between individuals. Subsequently, the systolic thrust was defined based on previously described physiological patterns in the apical–basal direction (red curve) (*Figure 2*).^11,12^ The systolic thrust is the first positive part of the red curve, encompassing the propulsive phase of systole during which the longitudinal force is directed towards the LV base. In this phase, the apical–basal impulse and systolic force vector angle are measured. The apical–basal impulse encompasses the area under the curve and reflects the magnitude of HDF during this phase of the cardiac cycle. The apical–basal impulse is also corrected for LV volume and adjusted for gravity acceleration and fluid density, allowing comparison between individuals. The systolic force vector angle reflects the orientation of the HDF during the propulsive part of systole.

### CRT implantation

CRT implantation was performed with a standard approach, i.e. by insertion of the right atrial and right ventricular leads via the subclavian or cephalic veins. Before insertion of the LV lead, venography of the coronary sinus was performed. The LV pacing lead was introduced through an 8 Fr guiding catheter and positioned in a posterior or posterolateral branch of the coronary sinus, if possible. A posterior or lateral lead position was achieved in 77% of patients in whom accurate LV lead placement data were available. Ninety seven per cent of the implanted devices had defibrillator functionality. CRT recipients were followed up at regular intervals at the heart failure outpatient clinic where the devices were interrogated. Atrioventricular and interventricular delays were empirically set at 120–140 and 0 ms, respectively. CRT optimization occurred at the discretion of the treating physician.

### Statistical analysis

Continuous variables are presented as mean ± standard deviation when normally distributed and as median with interquartile range when not normally distributed. Categorical variables are presented as numbers with percentages. Independent, Student’s *t*-tests and Mann–Whitney *U* tests were used to compare baseline characteristics between patients with and without LV reverse remodelling after six months of CRT.

Binary logistic regression analysis was performed to assess the association of baseline HDF parameters with LV reverse remodelling at six months. Variables that were significantly associated on univariable analysis (*P* < 0.05) were entered into the multivariable logistic regression models. Likelihood ratio testing was performed to test the incremental value of HDF parameters over conventional clinical, electrocardiographic, and echocardiographic parameters. Sensitivity analysis was performed to confirm the association of HDF parameters with LV reverse remodelling excluding patients with significant aortic valve disease because the presence of aortic valve disease could potentially influence the HDF analysis.

Delta (Δ) values were calculated for the QRS duration, LV volumetric, and HDF parameters. The Δ value was calculated as the (value at 6 months—value at baseline)/value at baseline ∗ 100 and indicates the percentage change compared with the baseline value. ΔQRS duration and ΔQRS axis were calculated from the values before and after CRT implantation. Pearson correlation coefficients were used to assess the correlation between ΔQRS, ΔLV volumetric parameters, and ΔHDF parameters.

Repeated measurements ANOVA was used to compare the change of different parameters over time between LV responders and LV non-responders.

Fifteen echocardiographic data sets were randomly selected for the evaluation of intra- and inter-observer variability of HDF parameters, using intraclass correlation coefficients. Excellent agreement was defined by an intraclass correlation coefficient > 0.90, whereas good agreement was defined by a value between 0.75 and 0.90. All tests were two-sided and *P* values <0.05 were considered statistically significant. All analyses were performed using SPSS Statistics for Windows, version 25.0 (IBM, Armonk, New York, USA).

## Results

### Study population

In total, 196 patients with heart failure and reduced LVEF were included in this analysis (see Supplementary data online, *Figure S1*). An ischaemic aetiology of heart failure was the most prevalent exclusion criterium. The mean age was 64 ± 11 years and 122 (62%) patients were male. LV reverse remodelling, defined by a ≥ 15% reduction of the LVESV, was present in 136 (69%) patients (LV responders). The cumulative overall survival rates for the overall population were 98%, 94%, 81%, and 64% at 1, 2, 5, and 10 year follow-up, respectively. Baseline clinical and echocardiographic characteristics before CRT implantation are listed in *Table 1*. There was no difference in symptoms, exercise capacity or quality of life between LV responders and LV non-responders before CRT implantation (baseline). Medical therapy for heart failure (beta-blockers and renin angiotensin aldosterone system blockers) was similar between the two patient groups. Combination therapy employing three heart failure drugs was infrequent, but not different among the two patient groups. LV non-responders were more frequently treated with loop diuretics when compared with LV responders (53 (88.3%) vs. 100 (73.5%) patients, *P* = 0.021). At baseline echocardiography, LV responders and LV non-responders had similar LV volumes and LVEF, but LV GLS was significantly higher in LV responders, compared with LV non-responders (7.8% vs. 6.2%, *P* = 0.001). Moreover, the left atria in LV non-responders were more dilated as compared with LV responders (45.6 mL/m² vs. 38.1 mL/m², *P* = 0.006). When evaluating HDF parameters before CRT implantation, there was no significant difference in HDF parameters reflecting the magnitude of HDF in the longitudinal and transverse directions (i.e. apical–basal strength and lateral–septal strength) in the complete heart cycle. However, the force vector angle, reflecting the orientation of HDF, was significantly higher in LV responders as compared with LV non-responders (67.1° vs. 64.1°, *P* = 0.002), indicating that the orientation of HDF at baseline is more severely misaligned with respect to the apex–base direction in LV non-responders than in LV responders. Finally, during the systolic thrust, the magnitude of the apical–basal HDF, represented by the apical–basal impulse, was higher in LV responders than in LV non-responders, reflecting a greater magnitude of apical–basal HDF during the propulsive part of systole in LV responders. Also, the orientation of the HDF during the systolic thrust, represented by the systolic force vector angle, was better aligned with the apex–base direction in LV responders vs. LV non-responders, before CRT implantation.

**Table 1:** Baseline clinical and echocardiographic characteristics

Variable	Overall (*n* = 196)	LV responders (*n* = 136)	LV non-responders (*n* = 60)	*P* value
Clinical characteristics				
Age, years	63.8 ± 10.5	64.2 ± 9.5	63.0 ± 12.7	0.448
Male sex, *n* (%)	122 (62.2%)	85 (62.5%)	37 (61.7%)	0.912
Body mass index, kg/m²	26.1 ± 4.6	26.1 ± 4.9	26.0 ± 4.0	0.868
NYHA III or IV, *n* (%)	128 (65.6%)	87 (64.0%)	41 (69.5%)	0.456
Quality of life	29.0 (16.0, 44.5)	30 (15.0, 44.0)	27 (17.5, 24.3)	0.713
6MWD, m	368.0 ± 117.6	365.2 ± 114.2	375.3 ± 127.5	0.648
ECG variables				
Baseline rhythm				
Sinus rhythm, *n* (%)	180 (91.8%)	128 (94.1%)	52 (86.7%)	0.079
Atrial fibrillation, *n* (%)	15 (7.7%)	7 (5.1%)	8 (13.3%)	**0**.**047**
Pacemaker, *n* (%)	1 (0.5%)	1 (0.7%)	0 (0.0%)	0.694
QRS duration, ms	166.3 ± 20.2	167.0 ± 18.0	164.8 ± 24.6	0.485
QRS axis, °	−26.0 (−47.5, 7.0)	−26.0 (−47.0, 6.5)	−28.0 (−48.0, 13.0)	0.823
Medication				
Combination of 3 HF therapies	70 (35.7%)	49 (36.0%)	21 (35.0%)	0.890
Beta-blocker, *n* (%)	160 (81.6%)	109 (80.1%)	51 (85.0%)	0.419
ACE-I/ARB, *n* (%)	178 (90.8%)	125 (91.9%)	53 (88.3%)	0.424
Loop diuretic, *n* (%)	153 (78.1%)	100 (73.5%)	53 (88.3%)	**0**.**021**
MRA, *n* (%)	92 (46.9%)	63 (46.3%)	29 (48.3%)	0.795
Echocardiographic characteristics				
LV end-diastolic volume, mL	214.8 ± 80.4	211.5 ± 70.8	222.3 ± 98.9	0.385
LV end-systolic volume, mL	162.1 ± 66.9	158.9 ± 60.0	169.3 ± 80.8	0.317
LV ejection fraction, %	25.3 ± 6.3	25.7 ± 6.4	24.5 ± 5.9	0.228
LV global longitudinal strain, %	7.2 ± 3.0	7.8 ± 3.0	6.2 ± 3.0	**0**.**001**
Left atrial volume index, mL/m²	40.3 ± 16.9	38.1 ± 15.5	45.6 ± 19.1	**0**.**006**
Significant MR, *n* (%) or severe MR	78 (42.2%)	52 (40.3%)	26 (46.4%)	0.439
Significant AR, *n* (%)	18 (9.2%)	9 (6.6%)	9 (15.0%)	0.061
Significant AS, *n* (%)	6 (3.1%)	2 (1.5%)	4 (6.7%)	0.052
Haemodynamic force parameters in the complete heart cycle				
Apical–basal strength, %	4.8 (3.5, 6.3)	4.9 (3.6, 6.6)	4.4 (3.3, 5.7)	0.085
Lateral–septal strength, %	1.5 (1.1, 2.0)	1.5 (1.1, 2.0)	1.7 (1.2, 2.2)	0.321
Force vector angle, °	66.2 ± 6.1	67.1 ± 5.8	64.1 ± 6.4	**0**.**002**
Haemodynamic force parameters in the systolic thrust				
Apical–basal impulse, %	4.8 (3.3, 6.5)	5.0 (3.8, 6.6)	3.4 (2.8, 6.1)	**0**.**012**
Systolic force vector angle, °	74.0 (70.0, 78.0)	74.0 (71.0, 78.0)	71.0 (65.0 76.0)	**<0**.**001**

Bold values represent significant *P* values (<0.05).

6MWD, 6 min walking distance; ACE-I, angiotensin converting enzyme inhibitor; AR, aortic regurgitation; ARB, angiotensin receptor blocker; AS, aortic stenosis; LV, left ventricular; MR, mitral regurgitation; MRA, mineralocorticoid receptor antagonist; NYHA, New York Heart Association class.

### Association of baseline HDF parameters with LV reverse remodelling

**Figure 3:**
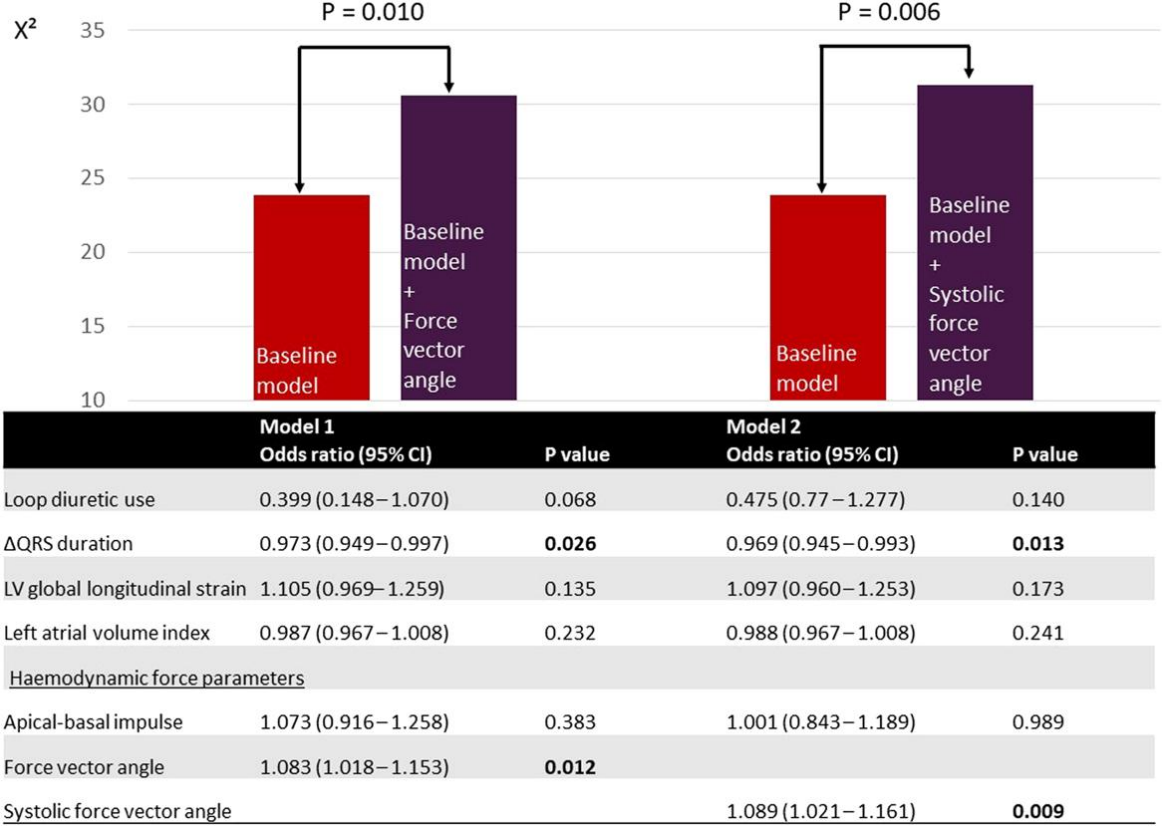
Multivariable logistic regression for the association with LV reverse remodelling and likelihood ratio test. CI, confidence interval; LV, left ventricular. Baseline model, loop diuretic use, ΔQRS duration, LV global longitudinal strain, left atrial volume index, apical–basal impulse. Bold values indicate significant *P* values (<0.05).

Univariable logistic regression was performed to explore the potential association of baseline variables with LV reverse remodelling (LV response). The results are presented in Supplementary data online, *Table S1*. Loop diuretic use [OR 0.367 (95% CI: 0.153, 0.880); *P* = 0.025], ΔQRS duration [OR 0.972 (95% CI: 0.951, 0.993), *P* = 0.011], LV GLS [OR 1.194 (95% CI: 1.069, 1.334); *P* = 0.002], left atrial volume index [OR 0.975 (95% CI: 0.957, 0.993), *P* = 0.008], the force vector angle in the complete heart cycle [OR 1.086 (95% CI: 1.030, 1.146); *P* = 0.002], the apical–basal impulse during the systolic thrust [OR 1.151 (95% CI: 1.012, 1.309); *P* = 0.033], and the systolic force vector angle in the systolic thrust [OR 1.085 (95% CI: 1.035,1.138); *P* = 0.001] were all significantly associated with LV response. The results of the multivariable logistic regression are presented in *Figure 3*. To minimize collinearity effects, the force vector angle in the complete heart cycle and the systolic force vector angle in the systolic thrust were included in separate regression models. The first model included the force vector angle in the complete heart cycle, while the second model included the systolic force vector angle. In the first model, ΔQRS duration [OR 0.973 (95% CI: 0.949–0.997), *P* = 0.026] and the force vector angle of HDF in the complete heart cycle [OR 1.083 (95% CI: 1.018, 1.153), *P* = 0.012] were independently associated with LV response. In the second model, ΔQRS duration [OR 0.969 (95% CI: 0.945–0.993), *P* = 0.013] and the systolic force vector angle [OR 1.089 (95% CI: 1.021, 1.161), *P* = 0.009] remained independently associated with LV response. The likelihood ratio test showed that the addition of the force vector angle (*P* = 0.010) or the systolic force vector angle (*P* = 0.006) to the baseline model provided incremental value over the baseline model including loop diuretic use, ΔQRS duration, LV GLS, left atrial volume index, and the apical–basal impulse. Sensitivity analysis (excluding 22 patients with significant aortic valve disease) confirmed the independent association of the force vector angle and the systolic force vector angle with LV reverse remodelling after CRT (see Supplementary data online, *Table S2*).

### Correlation between delta QRS, delta LV volumetric parameters, and delta HDF parameters

**Figure 4:**
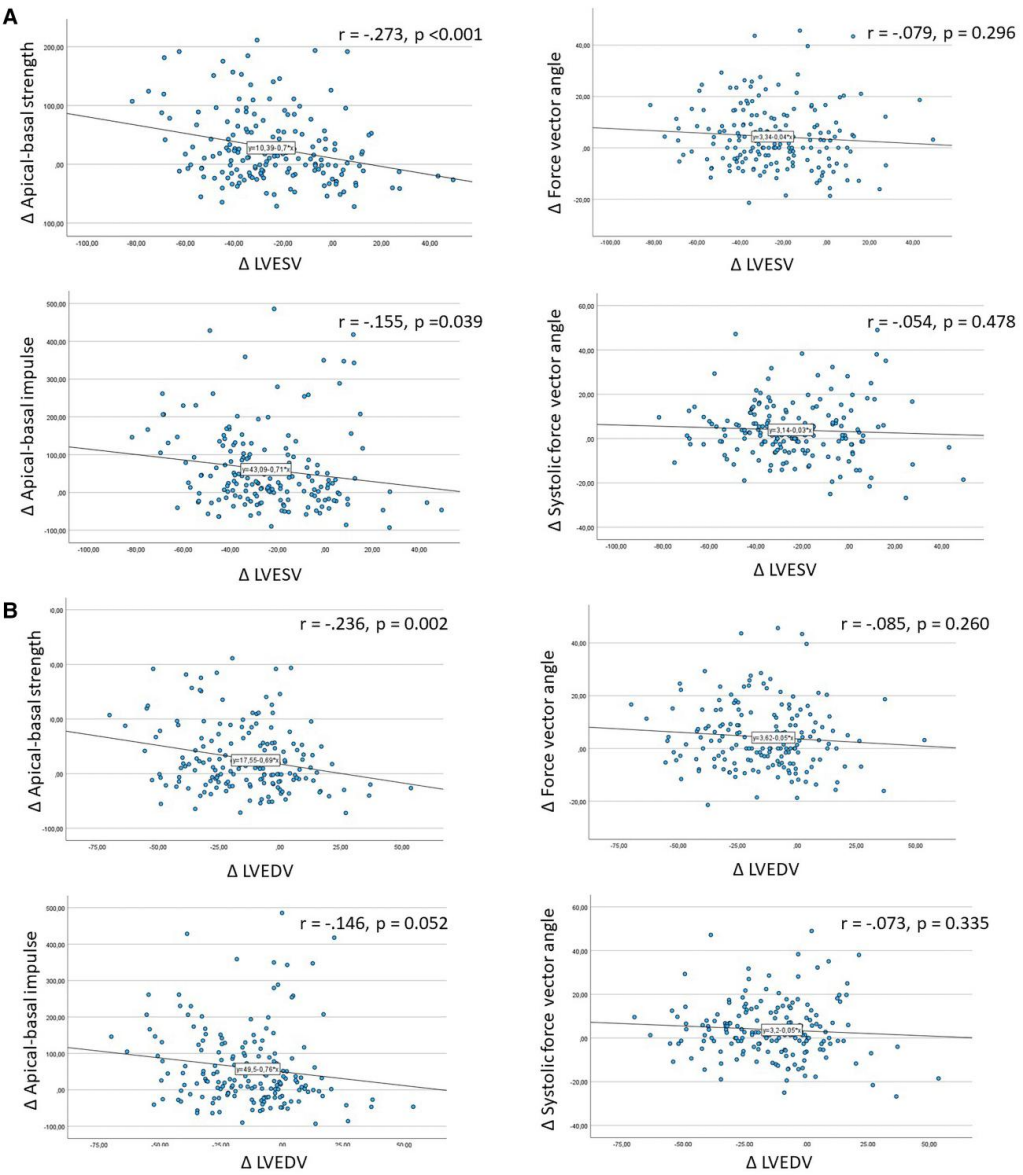

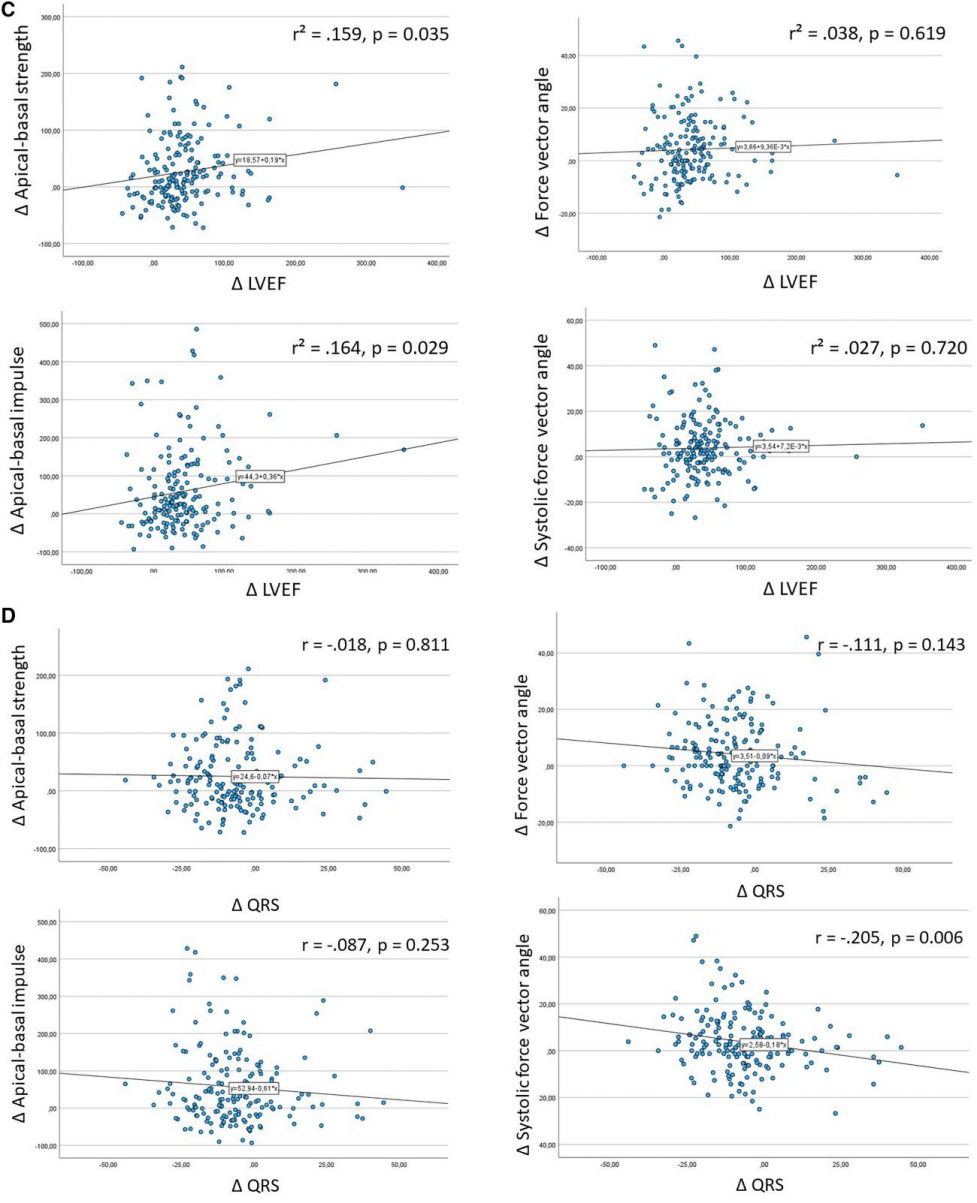
Correlation between ΔLVESV, ΔLVEDV, ΔLVEF, and ΔQRS with delta HDF parameters. (*A*) ΔLVESV. (*B*) ΔLVEDV. (*C*) ΔLVEF. (*D*) ΔQRS.

The scatter plots representing the correlation between ΔLVESV, ΔLV end-diastolic volume (LVEDV) ΔLVEF and ΔQRS duration with the Δ of the HDF parameters are shown in *Figure 4*. The correlation coefficients showed a weak correlation between ΔLVESV and ΔLVEF with Δapical–basal strength and Δapical–basal impulse while ΔLVEDV was only weakly correlated with Δapical–basal strength. ΔLVESV, ΔLVEDV, and ΔLVEF were not correlated with Δforce vector angle or Δsystolic force vector angle. ΔQRS duration showed a weak correlation with Δsystolic force vector angle. There was no correlation between ΔQRS axis and the Δ of the HDF parameters.

### Changes in HDFs after CRT in LV responders vs. LV non-responders

**Figure 5:**
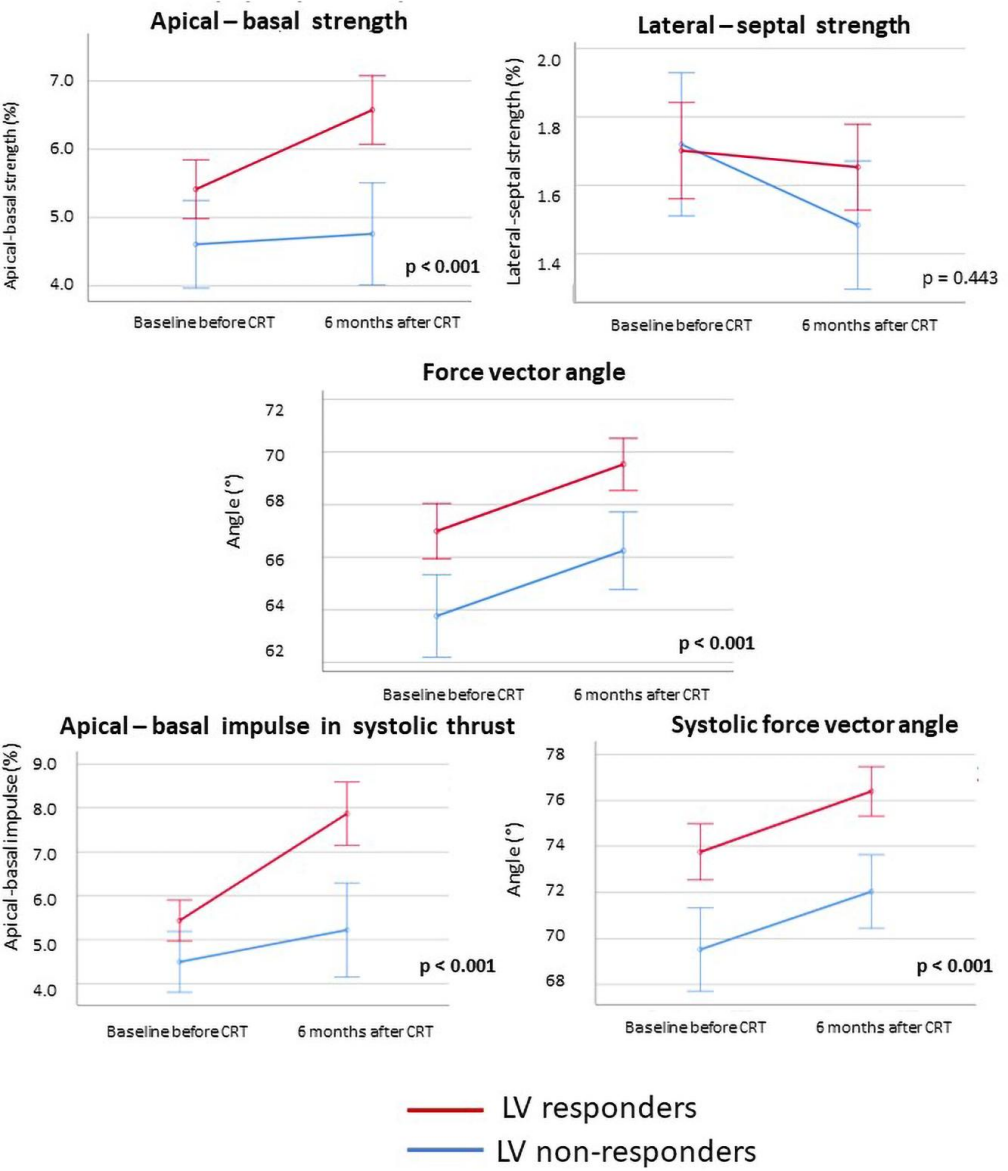
Evolution of HDF parameters after CRT implantation according to LV response. CRT, cardiac resynchronization therapy; HDF, haemodynamic force; LV, left ventricular. *P* values report the significance level for the F-test by repeated measurements ANOVA. Error bars indicate 95% confidence intervals.

The changes in clinical and echocardiographic characteristics from baseline to six months after CRT implantation in LV responders vs. LV non-responders are presented in *Table 2*. Both patient groups displayed a clinical response after CRT, with improvement in symptoms (New York Heart Association class), quality of life, and 6 min walking distance. In LV responders, there was more QRS shortening (*P* = 0.008) and a higher percentage of biventricular pacing (*P* = 0.032). In addition, LV responders showed significant LV reverse remodelling, with a significant reduction in LV end-systolic volume. Furthermore, a significant reduction in LV end-diastolic volume with a significant increase in LVEF and LV GLS was noted. Also, a significant reduction in mitral regurgitation occurred. Although LV non-responders also had a significant improvement in LVEF and LV GLS, they did not show a decrease in LV end-systolic volume or a significant reduction in mitral regurgitation, while a further increase in LV end-diastolic volume (continued LV dilatation) was noted in this group. The changes in HDF parameters after CRT implantation in LV responders and LV non-responders are shown in *Figure 5* and *Table 2*, while the absolute differences in HDF parameters are shown in Supplementary data online, *Table S3*. In the complete heart cycle, there was a significant increase in apical–basal strength in LV responders, which was absent in LV non-responders [4.9–6.0% in LV responders (*P* < 0.001) vs. 4.5–4.5% in LV non-responders (*P* = 0.691)]. This finding confirms the correlation between ΔLVESV and Δapical–basal strength. Conversely, both LV responders and LV non-responders encountered a significant increase in the force vector angle of HDF [67.1–69.9° in LV responders (*P* < 0.001) vs. 64.1–66.4° in LV non-responders (*P* = 0.014)], indicating a substantial change in the orientation of HDF and improved alignment of HDF with the apex–base direction in both patient groups. This finding confirms to the absence of a correlation between ΔLVESV with Δforce vector angle. However, the orientation of HDF at baseline was worse in LV non-responders. During the systolic thrust, the positive changes in the magnitude and orientation of HDF only occurred in LV responders. The magnitude of apical–basal HDF, represented by the apical–basal impulse, showed a significant increase in LV responders while no significant change occurred in the magnitude of apical–basal HDF in LV non-responders [5.0–7.0% in LV responders (*P* < 0.001) vs. 3.4–4.7% in LV non-responders (*P* = 0.084)]. The systolic force vector angle increased significantly in LV responders and did not improve significantly in LV non-responders, indicating that a positive change in the orientation of HDF during the propulsive phase of systole only occurred in LV responders [73.8–76.5° in LV responders (*P* < 0.001) vs. 71.0–72.1° in LV non-responders (*P* = 0.081)].

**Table 2:** Changes in clinical and echocardiographic parameters after 6 months of CRT

Variable	LV responders (*n* = 136)		*P* value	LV non-responders (*n* = 60)		*P* value
	Before CRT	After CRT		Before CRT	After CRT	
Clinical characteristics						
NYHA class	2.6 ± 0.7	1.8 ± 0.6	**<0**.**001**	2.7 ± 0.6	2.0 ± 0.8	**<0**.**001**
Quality of life	30.0 (15.0, 44.0)	12.5 (5.0, 21.0)	**<0**.**001**	27.0 (17.5, 47.3)	15.0 (5.0, 26.0)	**<0**.**001**
6MWD, m	366.5 ± 112.4	437.2 ± 101.4	**<0**.**001**	376.2 ± 130.8	410.6 ± 144.5	**0**.**001**
ECG characteristics						
QRS duration, ms	167.0 ± 18.0	152.3 ± 20.2	**<0**.**001**	164.8 ± 24.6	158.0 ± 23.3	0.071
QRS axis, °	−26.0 (−47.0, 6.5)	18.0 (−84.0, 216.0)	**<0**.**001**	−28.0 (−48.0, 13.0)	99.0 (−85.0, 200.0)	**<0**.**001**
ΔQRS duration, %	—	−8.4 ± 12.2		—	−2.6 ± 17.8	
Device characteristics						
Biventricular pacing, %	—	100 (98, 100)		—	99 (97, 100)	
Echocardiographic characteristics						
LVEDV, mL	211.5 ± 70.8	159.4 ± 58.2	**<0**.**001**	222.3 ± 98.9	232.9 ± 110.6	**0**.**030**
LVESV, mL	158.9 ± 60.0	100.1 ± 43.4	**<0**.**001**	169.3 ± 80.8	174.4 ± 91.8	0.151
LVEF, %	25.7 ± 6.4	38.4 ± 8.4	**<0**.**001**	24.5 ± 5.9	26.2 ± 6.9	**0**.**030**
LV GLS, %	7.8 ± 3.0	10.6 ± 3.7	**<0**.**001**	6.2 ± 3.0	7.1 ± 3.6	**0**.**008**
Significant MR, *n* (%)	52 (40.3%)	24 (17.6%)	**<0**.**001**	26 (46.4%)	18 (30.0%)	0.626
Haemodynamic force parameters in the complete heart cycle						
Apical–basal strength, %	4.9 (3.6, 6.6)	6.0 (4.6, 7.7)	**<0**.**001**	4.5 (3.3, 5.7)	4.5 (3.2, 5.8)	0.691
Lateral–septal strength, %	1.5 (1.1, 2.0)	1.5 (1.0, 2.1)	0.543	1.7 (1.2, 2.2)	1.3 (1.1, 1.6)	**0**.**048**
Force vector angle, °	67.1 ± 5.8	69.6 ± 5.4	**<0**.**001**	64.1 ± 6.4	66.4 ± 6.1	**0**.**014**
Haemodynamic force parameters in the systolic thrust						
Apical–basal impulse, %	5.0 (3.8, 6.6)	7.0 (4.4, 9.6)	**<0**.**001**	3.4 (2.8, 6.1)	4.7 (2.9, 7.0)	0.084
Systolic force vector angle, °	74.0 (71.0, 78.0)	78.0 (73.0, 80.0)	**<0**.**001**	71.0 (64.0, 76.0)	74.0 (67.5, 77.0)	0.081

Bold values represent significant *P* values (<0.05).

6MWD, 6 min walking distance; HDF, haemodynamic force; LVEDV, left ventricular end-diastolic volume; LVEF, left ventricular ejection fraction; LVESV, left ventricular end-systolic volume; LV GLS, left ventricular global longitudinal strain; MR, mitral regurgitation; NYHA, New York Heart Association class.

### Association between HDF parameters and LV super response

Additionally, LV super responders were compared with non-LV super responders (defined by a presence or absence of a reduction in LVESV ≥ 30% at six months). In total, 85 (43%) patients were LV super responders. Baseline clinical, electrocardiographic, and echocardiographic characteristics are presented in Supplementary data online, *Table S4*. LV super responders were more often female and were less symptomatic (according to New York Heart Association class) as compared with non-LV super responders. On echocardiography, LV GLS was more impaired and left atrial volume index was more enlarged in non-LV super responders vs. LV super responders. HDF analysis showed a significantly higher force vector angle at baseline in LV super responders vs. LV non-super responders. Univariable logistic regression showed an association between sex, New York Heart Association class, ΔQRS duration, LV GLS, left atrial volume index, and the force vector angle with LV super response (see Supplementary data online, *Table S5*). Multivariable logistic regression showed only an independent association of New York Heart Association class and ΔQRS duration with LV super response (see Supplementary data online, *Table S6*).

The changes in HDF parameters in LV super responders vs. LV non-super responders are presented in Supplementary data online, *Table S7* and Supplementary data online, *Figure S2*. LV super responders showed a significant increase in apical–basal strength, force vector angle, apical–basal impulse, and systolic force vector angle, whereas LV non-super responders only showed a significant increase in force vector angle and apical–basal impulse.

### Reproducibility of HDF analysis

The intra- and inter-observer reproducibility of HDF parameters is summarized in Supplementary data online, *Table S8*. The agreement was excellent for the HDF parameters in the complete heart cycle, the apical–basal impulse in the systolic thrust (inter-observer), and the force vector angle in the systolic thrust (intra-observer). The agreement was good for the apical–basal impulse in the systolic thrust (intra-observer) and the systolic force vector angle (inter-observer).

## Discussion

The main findings of the current study can be summarized as follows: (i) the orientation of HDF at baseline (represented by the force vector angle) was independently associated with LV reverse remodelling at six months after CRT; (ii) the force vector angle and the systolic force vector angle provided incremental value in the prediction of LV response over conventional clinical, electrocardiographic, and echocardiographic parameters; and (iii) after six months of CRT, the orientation of HDF (force vector angle) improved in both and LV non-responders, but the magnitude of apical–basal HDF (apical–basal strength and apical–basal impulse) only improved in LV responders.

### Association of baseline HDFs with LV reverse remodelling at 6 months after CRT implantation

The orientation of HDF, represented by the force vector angle, reflects the dominant direction of HDF during the complete heart cycle.^11^ The preferred direction of HDF is towards the LV base. On the polar histogram (*Figure 1*), perfect alignment of HDF with the apex–base direction corresponds to a force vector angle of 90°. Especially during the propulsive phase of systole (the systolic thrust), the direction of the HDF should constantly be directed at the LV base. Accordingly, the systolic force vector angle (representing the orientation of HDF during the systolic thrust) should come closer to 90° than the force vector angle of the complete heart cycle.

In patients with heart failure, reduced LVEF, and left bundle branch block, the direction or orientation of HDF is misaligned. The dominant force vector angle is directed away from the LV base (66.2° ± 6.1° in the current study population). This misalignment is caused by the presence of transverse HDF (represented by the blue curve in *Figure 1*), originating from reduced myocardial contractility and the existence of mechanical dyssynchrony. The current study showed that the degree of misalignment at baseline is worse in LV non-responders when compared with LV responders (force vector angle of 64.1° in LV non-responders vs. 67.1° in LV responders). This observation already suggests that the degree of misalignment of HDF may be associated with the occurrence of LV reverse remodelling after CRT implantation. Whether the orientation of HDF at baseline can indicate in which patients LV reverse remodelling will occur has not been evaluated previously. In the current study, regression analysis showed that the baseline force vector angle in the complete heart cycle and during systole remained independently associated with LV reverse remodelling after adjusting for established electrocardiographic markers (ΔQRS duration) and echocardiographic markers associated with LV reverse remodelling (LV GLS and left atrial volume index).^13,14^ Moreover, the force vector angle and systolic force vector angle provided incremental value over these conventional parameters. In other words, the worse the orientation of HDF at baseline, the less likely LV reverse remodelling is to occur after CRT implantation. Since factors predictive of LV reverse remodelling may risk stratify patients before CRT implantation, the orientation of HDF at baseline may contribute to predicting outcomes after CRT.

### Changes in HDFs after six months of CRT and LV reverse remodelling

After six months of CRT, changes in HDF occurred in both LV responders and LV non-responders.

First, the orientation of HDF improved in both LV responders and LV non-responders, represented by a significant improvement of the force vector angle in the complete heart cycle. Although the orientation of HDF at baseline was worse in LV non-responders, the reversal of mechanical dyssynchrony following biventricular pacing impacts on the orientation of HDF in both LV responders and LV non-responders.

Secondly, the magnitude of apical–basal HDF only improved in LV responders. The apical–basal strength in the complete heart cycle and the apical–basal impulse in the systolic thrust improved in LV responders while the apical–basal strength and impulse remained unchanged in LV non-responders. This finding suggests that an increase in the magnitude of apical–basal HDF is directly related to LV reverse remodelling (LV response), as seen in the scatter plots (*Figure 4A*) representing the correlation between Δapical–basal strength and ΔLVESV. Changes in HDF most likely occur before LV reverse remodelling takes place.^11^ Therefore, the findings of the current study suggest that an increase in magnitude of apical–basal HDF is necessary to induce LV reverse remodelling after CRT implantation.

### Clinical implications

The ability to calculate HDF on routinely acquired transthoracic echocardiographic images allows HDF to be used in clinical practice. Alterations in HDF occur before changes in LV myocardial deformation take place. Therefore, HDF may serve as a very sensitive marker of LV function and LV remodelling. Furthermore, the orientation of HDF is associated with the occurrence of LV reverse remodelling after CRT. Accordingly, the orientation of HDF at baseline may improve risk stratification of patients with heart failure eligible for CRT.

### Study limitations

The current study concerns a retrospective, monocentric analysis. The study population was carefully selected, including only patients with non-ischaemic cardiomyopathy and left bundle branch block because this study served as a proof of concept, including a study population in which possible confounders of the HDF analysis were minimized. Accordingly, selection bias might be present and might account for the increased number of LV responders to CRT. The findings of this study cannot be generalized to all patients with heart failure eligible for CRT. Further studies should include patients with ischaemic cardiomyopathy or right bundle branch block to evaluate the effect of these phenomena on HDF parameters.

Furthermore, the software uses the left ventricular outflow tract area and the mitral valve area as inflow and outflow area, respectively. Although the software accounts for a limited degree of mitral valve regurgitation, currently it was not possible to assess the influence of significant mitral valve regurgitation on the HDF analysis. While we focused HDF analysis on the complete heart cycle and the systolic thrust, future studies should include diastolic HDF parameters. It is also known that other factors, such as the extent of scar tissue in the LV and the underlying aetiology of heart failure, may influence the response to CRT. Cardiac magnetic resonance imaging and genetic tests were not routinely performed on the patients included in this study. Therefore, no systematic data are available to investigate the impact of scar tissue or genetic mutations.

Finally, HDF analysis should be performed on a validation cohort to confirm the results of this study.

## Conclusion

Echocardiography-derived HDF analysis in CRT recipients with non-ischaemic cardiomyopathy and left bundle branch block revealed that the orientation of HDF pre-implantation is associated with the occurrence of LV reverse remodelling after CRT in this carefully selected cohort of CRT recipients. While both LV responders and LV non-responders experienced haemodynamic benefit from CRT, only responders showed improvement in the magnitude of apical–basal HDF, indicating that LV reverse remodelling is closely related to the increase of the magnitude of apical–basal HDF. HDF analysis provides mechanistic insight in the haemodynamic changes after CRT. Further studies should include patients with ischaemic aetiology or non-left bundle branch block to test whether HDF analysis could improve the selection of patients who are likely to respond to CRT.

## Supplementary data

Supplementary data are available at *European Heart Journal - Cardiovascular Imaging* online.

## Supplementary Material

jeae181_Supplementary_Data

## Data Availability

The data underlying this article will be shared on reasonable request to the corresponding author.
