# Supplementary material for: Association between echocardiography-derived haemodynamic force parameters and left ventricular reverse remodelling after cardiac resynchronization therapy
Source: Eur Heart J Cardiovasc Imaging. 2024 Jul 17;25(12):1721–33. doi: 10.1093/ehjci/jeae181 (PMC11601885; doi:10.1093/ehjci/jeae181)
Supplement: jeae181_Supplementary_Data [file jeae181_supplementary_data.docx]

**Supplemental tables and figures**

**Supplemental table 1. Univariable logistic regression analysis for the association with LV response**

| Variable | Odds ratio (95% confidence interval) | p value |
| --- | --- | --- |
| Age | 1.011 (0.983, 1.040) | 0.447 |
| Male sex | 0.965 (0.516, 1.804) | 0.965 |
| Body mass index | 1.006 (0.941, 1.074) | 0.867 |
| Arterial hypertension | 0.733 (0.396, 1.357) | 0.324 |
| Diabetes mellitus | 1.121 (0.464, 2.709) | 0.800 |
| NYHA class III or IV | 0.779 (0.405, 1.501) | 0.456 |
| Quality of life | 0.996 (0.979, 1.013) | 0.635 |
| 6 MWD | 0.999 (0.996, 1.002) | 0.645 |
| Hemoglobin | 1.196 (0.854, 1.674) | 0.298 |
| eGFR | 1.006 (0.993, 1.020) | 0.388 |
| Beta-blocker use | 0.712 (0.312, 1.625) | 0.420 |
| ACE-I/ARB use | 1.501 (0.552, 4.082) | 0.426 |
| MRA use | 0.923 (0.502, 1.695) | 0.795 |
| Loop diuretic use | 0.367 (0.153, 0.880) | **0.025** |
| Heart rhythm | 0.503 (0.194, 1.306) | 0.158 |
| Atrial fibrillation | 0.353 (0.122, 1.022) | 0.055 |
| Biventricular pacing at 6 months | 1.010 (0.989, 1.031) | 0.364 |
| QRS duration | 1.005 (0.990, 1.021) | 0.483 |
| ∆QRS duration | 0.972 (0.951, 0.993) | **0.011** |
| LV end-systolic volume | 0.998 (0.993, 1.002) | 0.316 |
| LV end-diastolic volume | 0.998 (0.995, 1.002) | 0.384 |
| LV ejection fraction | 1.030 (0.982, 1.081) | 0.227 |
| LV global longitudinal strain | 1.194 (1.069, 1.334) | **0.002** |
| Left atrial volume index | 0.975 (0.957, 0.993) | **0.008** |
| Significant mitral regurgitation | 0.960 (0.740, 1.244) | 0.756 |
| HDF parameters at baseline |  |  |
| Complete heart cycle |  |  |
| Apical-basal strength | 1.143 (0.984, 1.328) | 0.079 |
| Lateral-septal strength | 0.911 (0.619, 1.341) | 0.636 |
| Force vector angle | 1.086 (1.030, 1.146) | **0.002** |
| Systolic thrust |  |  |
| Apical-basal impulse | 1.151 (1.012, 1.309) | **0.033** |
| Systolic force vector angle | 1.085 (1.035, 1.138) | **0.001** |

Abbreviations: 6 MWD = six-minute walking distance; ACE-I/ARB = angiotensin converting enzyme-inhibitor/angiotensin receptor blocker; eGFR = estimated glomerular filtration rate; LV = left ventricular; MRA = mineralocorticoid receptor antagonist; NYHA = New York Heart Association. Bold values represent significant P values (<0.05).

**Supplemental Table 2. Sensitivity analysis, excluding significant aortic valve disease: multivariable logistic regression for the association with LV response**

| Variable | Odds ratio (95% CI) | P value | Odds ratio (95% CI) | P value |
| --- | --- | --- | --- | --- |
| Atrial fibrillation | 0.304 (0.074, 1.251) | 0.099 | 0.379 (0.085, 1.685) | 0.379 |
| ∆QRS duration | 0.972 (0.947, 0.999) | **0.039** | 0.968 (0.942, 0.994) | **0.017** |
| LV global longitudinal strain | 0.862 (0.751, 0.990) | **0.035** | 0.891 (0.773, 1.028) | 0.115 |
| Left atrial volume index | 0.993 (0.970, 1.018) | 0.594 | 0.994 (0.970, 1.019) | 0.656 |
| Force vector angle | 1.072 (1.006, 1.142) | **0.032** | ----------------------- | -------- |
| Systolic force vector angle | ---------------------------------- | -------- | 1.084 (1.020, 1.152) | **0.009** |

**Abbreviations:** CI = confidence interval; LV = left ventricular. Bold values indicate significant p values (<0.05).

**Supplemental table 3. Absolute difference in HDF parameters between baseline and 6 months after CRT**

| HDF parameter | LV responders  (N=136) | LV non-responders  (N=60) | P value |
| --- | --- | --- | --- |
| *Complete heart cycle* |  |  |  |
| Apical-basal strength, % | ↑1.2 ± 3.1 | ↑0.1 ± 2.4 | **0.027** |
| Lateral-septal strength, % | ↓-0.0 ± 0.9 | ↓-0.2 ± 0.9 | 0.226 |
| Force vector angle, ° | ↑2.5 ± 6.9 | ↑2.0 ± 8.5 | 0.655 |
| *Systolic thrust* |  |  |  |
| Apical-basal impulse, % | ↑2.4 ± 4.1 | ↑0.7 ± 3.1 | **0.007** |
| Systolic force vector angle, ° | ↑2.6 ± 8.0 | ↑2.1 ± 10.5 | 0.696 |

Abbreviations: HDF = haemodynamic force; LV = left ventricular. Bold values represent significant p values (<0.05).

**Supplemental Table 4. Baseline clinical and echocardiographic characteristics according to LV super responders, and LV non super responders**

| Variable | Overall  (N = 196) | LV Super responders  (N = 85) | LV Non super-responders (N = 111) | | p value | |
| --- | --- | --- | --- | --- | --- | --- |
| *Clinical characteristics* |  |  |  | |  | |
| Age, years | 63.8 ± 10.5 | 65.4 ± 8.9 | 62.6 ± 11.6 | | 0.071 | |
| Male sex, N (%) | 122 (62.2%) | 46 (54.1%) | 76 (68.5%) | | **0.040** | |
| Body mass index, kg/m² | 26.1 ± 4.6 | 26.4 ± 4.6 | 25.9 ± 4.6 | | 0.472 | |
| NYHA III or IV, N (%) | 128 (65.6%) | 48 (56.5%) | 80 (72.7%) | | **0.018** | |
| Quality of life | 29.0 (16.0, 44.5) | 28.5 (9.0, 42.5) | 31.0 (20.0, 47.0) | |  | |
| 6MWD, m | 368.0 ± 117.6 | 358.0 ± 114.5 | 377.1 ± 120.4 | | 0.329 | |
| *ECG variables* |  |  |  | |  | |
| Baseline rhythm  Sinus rhythm, N (%)  Atrial fibrillation, N (%)  Pacemaker, N (%) | 180 (91.8%) 15 (7.7%) 1 (0.5%) | 80 (94.1%)  4 (4.7%)  1 (1.2%) | 100 (90.1%) 11 (9.9%) 0 (0%) | | 0.213 0.307 0.278 0.434 | |
| QRS duration, ms | 166.3 ± 20.2 | 168.9 ± 17.2 | 164.3 ± 22.1 | | 0.115 | |
| QRS axis, ° | -26 (-48, 7) | -23 (-48, 13) | -29.5 (-47.0, 3.5) | |  | |
| *Medication* |  |  |  | |  | |
| Combination of 3 HF therapies | 70 (35.7%) | 34 (40.0%) | 36 (32.4%) | | 0.273 | |
| Beta-blocker, N (%) | 160 (81.6 %) | 72 (84.7%) | 88 (79.3%) | | 0.331 | |
| ACE-I/ARB, N (%) | 178 (90.8%) | 78 (91.8%) | 100 (90.1%) | | 0.687 | |
| Loop diuretic, N (%) | 153 (78.1%) | 67 (78.8%) | 86 (77.5%) | | 0.821 | |
| MRA, N (%) | 92 (46.9%) | 42 (49.4%) | 50 (45.0%) | | 0.544 | |
| *Echocardiographic characteristics* | | |  |  | |  |
| LV end-diastolic volume, ml | 214.8 ± 80.4 | 218.0 ± 72.1 | 212.3 ± 86.4 | | 0.627 | |
| LV end-systolic volume, ml | 162.1 ± 66.9 | 163.2 ± 60.6 | 161.1 ± 71.6 | | 0.828 | |
| LV ejection fraction, % | 25.3 ± 6.3 | 25.9 ± 6.7 | 24.9 ± 6.0 | | 0.304 | |
| LV global longitudinal strain, % | 7.2 ± 3.0 | 7.8 ± 3.1 | 6.8 ± 3.0 | | **0.021** | |
| Left atrial volume index, ml/m² | 40.3 ± 16.9 | 36.1 ± 14.7 | 43.7 ± 17.8 | | **0.002** | |
| Significant MR, N (%) **or severe MR** | 78 (42.2%) | 33 (40.2%) | 45 (43.7%) | | 0.637 | |
| *Haemodynamic force parameters in the complete heart cycle* | | | | | | |
| Apical-basal strength, % | 4.8 (3.5, 6.3) | 4.9 (3.5, 6.8) | 4.7 (3.4, 5.9) | | 0.136 | |
| Lateral-septal strength, % | 1.5 (1.1, 2.0) | 1.5 (1.1, 1.9) | 1.5 (1.1, 2.2) | | 0.460 | |
| Force vector angle, ° | 66.2 ± 6.1 | 67.1 ± 5.7 | 65.3 ± 6.4 | | **0.037** | |
| *Ha*  *emodynamic force parameters in the systolic thrust* | | | | | | |
| Apical-basal impulse, % | 4.8 (3.3, 6.5) | 4.9 (3.9, 6.5) | 4.5 (3.0, 6.4) | | 0.260 | |
| Systolic force vector angle, ° | 74.0 (70.0, 78.0) | 74.0 (71.3, 78.0) | 74.0 (67.3, 77.0) | | 0.126 | |

Abbreviations: 6 MWD = six-minute walking distance; ACE-I =angiotensin converting enzyme inhibitor; ARB = angiotensin receptor blocker; LV = left ventricular; MR = mitral regurgitation; MRA =mineralocorticoid receptor antagonist; NYHA = New York Heart Association class.
Bold values represent significant p values (<0.05).

**Supplemental Table 5. Univariable logistic regression for the association with LV super response**

| Baseline variable | Odds ratio (95% confidence interval) | p value |
| --- | --- | --- |
| Age | 1.026 (0.997, 1.056) | 0.075 |
| Male sex | 1.841 (1.026, 3.305) | **0.041** |
| Body mass index | 1.023 (0.962, 1.087) | 0.470 |
| Arterial hypertension | 1.316 (0.739, 2.342) | 0.351 |
| Diabetes mellitus | 0.689 (0.300, 1.581) | 0.379 |
| NYHA class III or IV | 0.486 (0.267, 0.886) | **0.019** |
| Quality of life | 0.990 (0.974, 1.006) | 0.216 |
| 6 MWD | 0.999 (0.996, 1.001) | 0.328 |
| Hemoglobin | 1.107 (0.811, 1.511) | 0.523 |
| eGFR | 1.009 (0.997, 1.022) | 0.154 |
| Beta-blocker use | 1.448 (0.685, 3.058) | 0.332 |
| ACE-I/ARB use | 1.226 (0.454, 3.308) | 0.688 |
| MRA use | 1.192 (0.676, 2.099) | 0.544 |
| Loop diuretic use | 1.082 (0.546, 2.146) | 0.821 |
| Heart rhythm | 0.719 (0.269, 1.922) | 0.551 |
| Atrial fibrillation | 0.449 (0.138, 1.463) | 0.184 |
| QRS duration | 1.011 (0.997, 1.026) | 0.116 |
| ∆QRS duration | 0.975 (0.954, 0.996) | **0.020** |
| LV end-diastolic volume | 1.001 (0.997, 1.004) | 0.625 |
| LV end-systolic volume | 1.000 (0.996, 1.005) | 0.827 |
| LV ejection fraction | 1.024 (0.979, 1.072) | 0.302 |
| LV global longitudinal strain | 1.119 (1.016, 1.231) | **0.022** |
| Left atrial volume index | 0.971 (0.952, 0.990) | **0.003** |
| Significant mitral regurgitation | 0.868 (0.482, 1.564) | 0.637 |
| HDF parameters |  |  |
| Complete heart cycle |  |  |
| Apical-basal strength | 1.090 (0.967, 1.228) | 0.159 |
| Lateral-septal strength | 0.937 (0.651, 1.347) | 0.724 |
| Force vector angle | 1.053 (1.003, 1.105) | **0.039** |
| Systolic thrust |  |  |
| Apical-basal impulse | 1.049 (0.943, 1.167) | 0.377 |
| Systolic force vector angle | 1.036 (0.992, 1.081) | 0.108 |

Abbreviations: 6 MWD = six-minute walking distance; ACE-I/ARB = angiotensin converting enzyme-inhibitor/angiotensin receptor blocker; eGFR = estimated glomerular filtration rate; LV = left ventricular; MRA = mineralocorticoid receptor antagonist; NYHA = New York Heart Association. Bold values represent significant p values (<0.05).

**Supplemental Table 6. Multivariable logistic regression analysis for association of covariates with
LV super response**

| Variable | Model 1 *Odds ratio (95% CI)* | p value |
| --- | --- | --- |
| Male sex | 1.673 (0.842, 3.326) | 0.142 |
| NYHA III or IV | 0.416 (0.208, 0.834) | **0.014** |
| ΔQRS duration | 0.976 (0.953, 0.999) | **0.041** |
| LV global longitudinal strain | 1.073 (0.960, 1.200) | 0.214 |
| Left atrial volume index | 0.984 (0.963, 1.005) | 0.130 |
| Force vector angle | 1.047 (0.990, 1.106) | 0.108 |

Abbreviations: CI = confidence interval; LV = left ventricular.; NYHA = New York Heart Association class
Bold values represent significant p values (<0.05).

**Supplemental Table 7. Changes in clinical and echocardiographic parameters after 6 months of CRT in LV super responders versus LV non super responders**

| Variable | LV super responders  (N = 85) | | p value | LV non super responders (N = 111) | | p value |
| --- | --- | --- | --- | --- | --- | --- |
|  | *Before CRT* | *After CRT* |  | *Before CRT* | *After CRT* |  |
| NYHA class | 2.5 ± 0.7 | 1.8 ± 0.7 | **<0.001** | 2.7 ± 0.6 | 1.9 ± 0.7 | **<0.001** |
| Quality of life | 28.5 (9.0, 42.5) | 12.0 (5.0, 26.0) | **<0.001** | 31.0 (20.0, 47.0) | 14.0 (6.0, 26.5) | **<0.001** |
| 6MWD, m | 358.0 ± 114.5 | 441.4 ± 105.9 | **<0.001** | 277.1 ± 120.4 | 424.3 ± 126.2 | **<0.001** |
| QRS duration, ms | 168.9 ± 17.2 | 152.6 ± 20.1 | **<0.001** | 164.3 ± 22.1 | 155.2 ± 22.2 | **<0.001** |
| QRS axis, | -23.0 (-48.0, 13.0) | 2 (-82.3, 213) | **<0.001** | -29.5 (-47.0, 3.5) | 93.0 (-85.0, 211.0) | **<0.001** |
| LVEDV, ml | 218.0 ± 72.1 | 144.6 ± 50.8 | **<0.001** | 212.4 ± 86.4 | 210.4 ± 94.3 | 0.524 |
| LVESV, ml | 163.2 ± 60.6 | 89.3 ± 39.0 | **<0.001** | 161.1 ± 71.6 | 145.5 ± 78.9 | **<0.001** |
| LVEF, % | 25.9 ± 6.7 | 39.6 ± 9.1 | **<0.001** | 24.9 ± 6.0 | 30.9 ± 8.6 | **<0.001** |
| LV GLS, % | 7.8 ± 3.1 | 11.1 ± 3.5 | **<0.001** | 6.8 ± 3.0 | 8.3 ± 3.9 | **<0.001** |
| Significant MR,  N (%) | 33 (40.2%) | 10 (12.2%) | **<0.001** | 45 (43.7%) | 32 (31.4%) | **0.023** |
| Apical-basal strength, % | 4.9 (3.5, 6.8) | 6.2 (5.0, 8.6) | **<0.001** | 4.7 (3.4, 5.9) | 4.9 (3.4, 6.5) | 0.148 |
| Lateral-septal strength, % | 1.5 (1.1, 1.9) | 1.5 (1.0, 2.2) | 0.655 | 1.5 (1.1, 2.2) | 1.4 (1.1, 1.8) | 0.079 |
| Force vector angle, ° | 67.1 ± 5.7 | 70.5 ± 5.4 | **<0.001** | 65.3 ± 6.4 | 67.1 ± 5.7 | **0.016** |
| Apical-basal impulse, % | 4.9 (3.9, 6.5) | 8.0 (5.0, 10.1) | **<0.001** | 4.5 (3.0, 6.4) | 5.6 (3.4, 7.9) | **0.003** |
| Systolic force vector angle, ° | 74.0 (71.3, 78.0) | 78.0 (74.0,  1.0) | **<0.001** | 74.0 (67.3, 77.0) | 74.0 (70.0, 79.0) | 0.071 |

Abbreviations: 6 MWD = six-minute walking distance; HDF = haemodynamic force; LVEDV = left ventricular end-diastolic volume; LVEF = left ventricular ejection fraction; LVESV = left ventricular end-systolic volume; LV GLS = left ventricular global longitudinal strain; MR = mitral regurgitation; NYHA = New York Heart Association class.
Bold values represent significant p values (<0.05).

**Supplemental table 8. Intra-observer and interobserver correlation coefficients for HDF parameters**

|  | Intraclass Correlation (95% CI) | p value |
| --- | --- | --- |
| *Intra-observer variability* |  |  |
| Apical-basal strength, % | 0.95 (0.87, 0.98) | **<0.001** |
| Lateral-septal strength, % | 0.96 (0.89, 0.98) | **<0.001** |
| Force vector angle, ° | 0.96 (0.89, 0.98) | **<0.001** |
| Apical-basal impulse, % | 0.85 (0.62, 0.94) | **<0.001** |
| Systolic force vector angle, ° | 0.90 (0.75, 0.96) | **<0.001** |
| *Inter-observer variability* |  |  |
| Apical-basal strength, % | 0.95 (0.88, 0.98) | **<0.001** |
| Lateral-septal strength, % | 0.96 (0.90, 0.98) | **<0.001** |
| Force vector angle, ° | 0.93 (0.83, 0.97) | **<0.001** |
| Apical-basal impulse, % | 0.96 (0.89, 0.98) | **<0.001** |
| Systolic force vector angle, ° | 0.81 (0.52, 0.92) | **<0.001** |

Abbreviations: AB = apical-basal; Bold values represent significant p values (<0.05).

**Supplemental Figure 1. Patient selection**

**
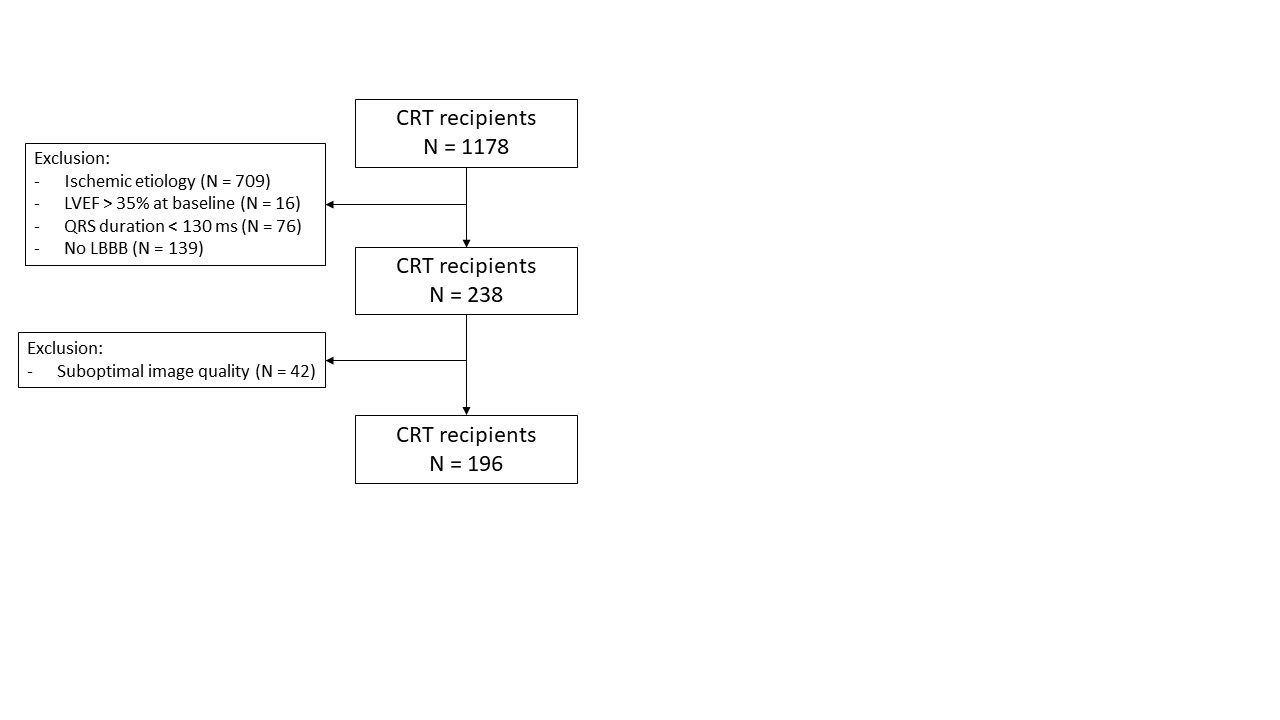
**

Abbreviations: LBBB = left bundle branch block; LVEF = left ventricular ejection fraction

**Supplemental Figure 2. Evolution of HDF parameters after CRT implantation according to LV super response**

**
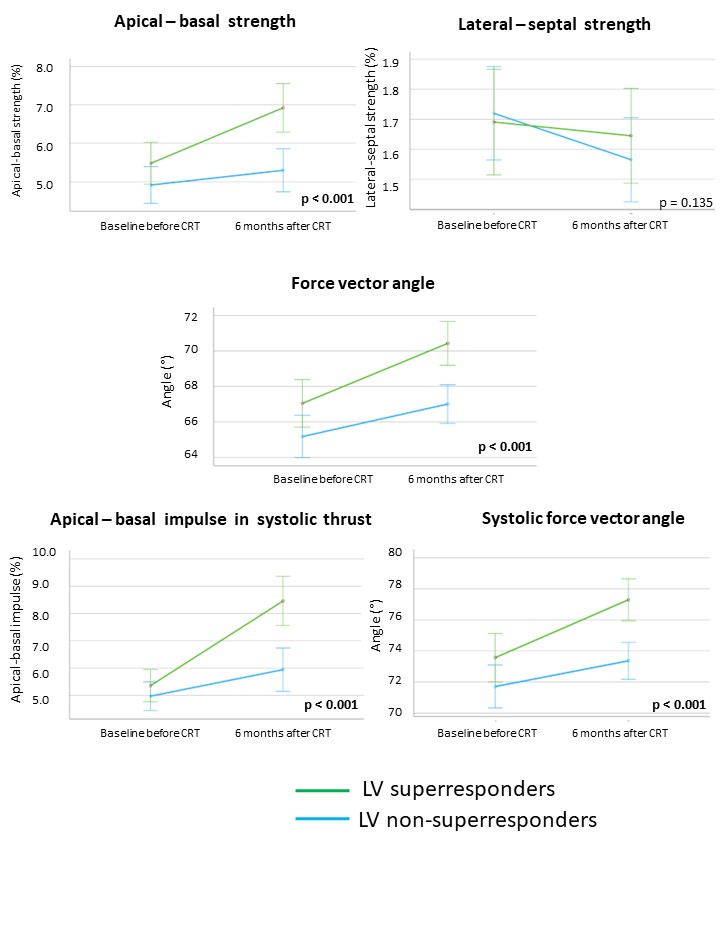
**

Abbreviations: CRT = cardiac resynchronization therapy; HDF = haemodynamic force; LV = left ventricular. P values report the significance level for the F-test by repeated measurements ANOVA. Bold values represent significant p values (<0.05). Error bars indicate 95% confidence intervals.
